# Altered avalanche dynamics in a developmental NMDAR hypofunction model of cognitive impairment

**DOI:** 10.1038/s41398-017-0060-z

**Published:** 2018-01-10

**Authors:** Saurav Seshadri, Andreas Klaus, Daniel E. Winkowski, Patrick O. Kanold, Dietmar Plenz

**Affiliations:** 1 Section on Critical Brain Dynamics, National Institute of Mental Health, Bethesda, MD USA; 2 Department of Biology, Univ. of Maryland, College Park, MD USA

## Abstract

Disturbed activity patterns in cortical networks contribute to the pathophysiology of schizophrenia (SZ). Several lines of evidence implicate NMDA receptor hypofunction in SZ, and blocking NMDA receptor signaling during early neurodevelopment produces cognitive deficits in rodent models that resemble those seen in schizophrenic patients. However, the altered network dynamics underlying these cognitive impairments largely remain to be characterized, especially at the cellular level. Here, we use in vivo two-photon calcium imaging to describe pathological dynamics, occurring in parallel with cognitive dysfunction, in a developmental NMDA receptor hypofunction model. We observed increased synchrony and specific alterations in spatiotemporal activity propagation, which could be causally linked to a previously unidentified persistent bursting phenotype. This phenotype was rescued by acute treatment with the NMDA receptor co-agonist D-serine or the GABA_B_ receptor agonist baclofen, which similarly rescued working memory performance. It was not reproduced by optogenetic inhibition of fast-spiking interneurons. These results provide novel insight into network-level abnormalities mediating the cognitive impairment induced by NMDA receptor hypofunction.

## Introduction

Network-level disturbances are thought to be a core pathological entity in schizophrenia (SZ)^1,2^. These disturbances are brought about by a combination of genetic and environmental risk factors, which act early in neurodevelopment to disrupt the trajectory of network formation. One of the most extensively studied of these risk factors is *N*-methyl D-aspartate receptor (NMDAR) hypofunction, due to the psychotomimetic effects of NMDAR antagonists^3^, consistent association of mutations in NMDAR signaling-related genes with SZ^4^, and successful reproduction of key SZ-associated phenotypes in NMDAR loss-of-function rodent models^5–7^. For example, both SZ patients^8,9^ and developmental NMDAR hypofunction model animals^10^ show reduced immunoreactivity for the fast-spiking interneuron marker parvalbumin (PV). At the level of cognition, genetic^11^ or pharmacological^12,13^ blockade of NMDAR signaling during development leads to impaired working memory, a symptom commonly seen in SZ patients^14,15^. Still, the precise nature of the network dysfunction mediating this effect remains unknown.

Investigation of cortical network dynamics in SZ has tended to use population measures, such as functional magnetic resonance imaging (fMRI) and electroencephalogram (EEG). These approaches have revealed significant changes in functional connectivity^16^ and oscillatory power^17,18^ respectively, some of which are reproduced in NMDAR hypofunction models^5,6^. A limitation of these methods is their temporal and spatial resolution, which prevents them from providing direct insight into activity propagation in local populations of neurons. However, such microcircuits must contribute to transforming molecular alterations into network-level dysfunction. Identifying pathological phenotypes at this level may therefore be important to understanding the role of NMDAR signaling in cortical development and functioning, which may in turn relate to disease. Recording activity with cellular resolution, using 2-photon imaging (2PI) of calcium indicators, is one way to fill in this gap in understanding, and has been applied recently to an adult NMDAR hypofunction model^19^. While this study advances our understanding of the altered network dynamics caused by NMDAR antagonism, our work is distinguished by several factors: first, we use a neurodevelopment-specific intervention, which may more accurately reproduce the etiology of cognitive impairment observed in SZ^20^; second, we use pharmacological rescue and behavioral validation to establish a link between dynamic phenotypes and cognitive impairment; and third, our analysis provides distinct, complementary insights by focusing on temporal propagation of neuronal activity rather than synchronous co-activation of neuronal ensembles. In the present study, we extend the 2PI approach to a validated developmental model of cognitive impairment^3^, and use theoretically motivated analyses to characterize and quantify dysfunctional higher-order interactions.

Over the past decade, the theory of criticality has gained support as a conceptual framework to describe the spatiotemporal organization of activity in cortex^21,22^. This theory predicts that cortical networks reside near a critical point, at which excitation and inhibition are balanced and spontaneous cortical activity takes the form of ‘neuronal avalanches’ (cascades of propagating activity governed by power laws^23^). Several aspects of network functioning are maximized by avalanche dynamics^24–26^, suggesting that they may contribute to cognitive functioning. Previous work from our group, showing that neuronal avalanches are altered by pharmacological manipulation of excitatory/inhibitory balance and dopaminergic signaling^27,28^ (both of which affect working memory performance^29,30^ and SZ pathophysiology^1^), led us to believe that avalanche dynamics may be a sensitive readout for changes in network dynamics associated with cognitive impairment. Importantly, avalanches have been recorded in a range of neural systems [from rats^27,31^ to non-human primates^32^ and humans^33–37^ using a range of techniques (2PI^31^, local field potential^27,32^, magnetoencephalogram^34,38^, fMRI^35,36^, ECoG, and EEG^37,39^)], suggesting potential for translation.

In the present study, we report significantly altered neuronal avalanches in a rodent model of cognitive impairment. This novel dynamic phenotype is induced by neurodevelopmental NMDAR antagonism, and is characterized by an inability to temporally restrict activity propagation within an avalanche.

## Methods

### Rodents

Female Sprague-Dawley rats (Taconic) were used for all drug treatment experiments. Female transgenic PV-Cre rats with Long-Evans background (NIDA IRP Transgenic Rat Project) were used for optogenetic experiments. Rats were 4-6 weeks old at the time of imaging or behavioral testing. All procedures were conducted in accordance with the institutional Animal Care and Use Committee. N for behavioral data was as follows: SAL group, 13 rats; PCP group, 6 rats; SAL + D-serine group, 5 rats; PCP + D-serine group, 6 rats. N for pharmacological treatment imaging data was as follows: SAL group, 15 movies from 5 rats; PCP group, 15 movies from 5 rats; SAL + D-serine group, 12 movies from 4 rats; PCP + D-serine group, 15 movies from 4 rats, PCP group 2 (baclofen treated), 12 movies from 4 rats, PCP + baclofen group, 12 movies from 4 rats. *N* for optogenetic stimulation imaging data in PV-Cre rats was as follows: Control (Ctrl) group, 20 movies from 7 rats; LED stimulation, 20 movies from 7 rats. Sample sizes (4–6 animals per group) were chosen based on similar studies^19^ without biasing for expected effect sizes, in order to obtain statistical significance without overpowering analysis to detect negligibly small effects.

### Drug treatment

Phencyclidine obtained from the NIH Veterinary Pharmacy was freshly dissolved in sterile saline to a final concentration of 5 mg/ml, filtered with a Millex-GV 0.22 µm PVDF membrane (Millipore), and administered to P7, P9, and P11 pups subcutaneously at 2 ml/kg (final dosage, 10 mg/kg). Littermates were randomly assigned to PCP or SAL treatment groups at P7. D-serine (Sigma) was dissolved in sterile saline to a final concentration of 200 mg/ml, filtered with a Millex-GV 0.22 µm PVDF membrane (Millipore), and administered to P35-42 rats intraperitoneally at 4 ml/kg (final dosage, 800 mg/kg). Behavioral testing or 2PI was conducted 30–60 min after D-serine injection. Baclofen (Sigma) was dissolved in sterile saline to a final concentration of 2.5 mg/ml, filtered with a Millex-GV 0.22 µm PVDF membrane (Millipore), and administered to P35-42 rats intraperitoneally at 2 ml/kg (final dosage, 5 mg/kg), 15 min before 2PI. Drug dosages were determined based on previous studies^7,40,41^.

### Viral gene expression

YC2.60 was subcloned from pRSET_B_ (see ref. 42) to pAAV-CaMKII (see ref. 43) using restriction enzymes BamHI and EcoRI (New England Biolabs). High-titer virus (1.3 × 10^13^ virus molecules/ml) was prepared by the UNC Vector Core. For inhibitory opsin expression, AAV-EF1a-DIO-eNpHR3.0-mCherry was obtained from the UNC Vector Core. Rats at P14-P19 were anesthetized, immobilized in a stereotaxic frame, and injected via a 34 G needle (Hamilton) with 2 µl of virus (1:1 ratio when injecting multiple viruses) at a depth of 250 µm in M1. Animals were given subcutaneous antibiotic (Baytril, 0.227%) and analgesic (Ketoprofen, 5 mg/kg) postoperatively. Imaging was performed at least 2 weeks after surgery to allow for recovery and YC2.60 or opsin expression.

### Acute in vivo 2-photon imaging

Cranial window implantation was carried out as previously described^31^. Briefly, P35-42 rats were anesthetized, immobilized in a stereotaxic frame, and an oval window was drilled into the skull, followed by removal of the dura, application of low-melting point agarose (Invitrogen), and fixation of a glass coverslip using dental cement (Dentsply). A custom-made head bar was attached to the contralateral skull surface using Metabond (Parkell). Animals were allowed to recover for at least 3 h following surgery before imaging with a Bergamo series 2-photon microscope (Thorlabs). For imaging, rodents were lightly anesthetized (1% isoflurane), wrapped in a homeothermic blanket (Harvard Apparatus), and head-fixed under a 20x objective (Olympus). Coarse focusing to find cells was done using LED excitation to minimize photodamage to the cortex. For recording, Ti:Sapphire laser (Coherent) excitation at 830 nm was used to image a 557 × 557 µm^2^ field at 512 × 512 resolution and 15 frames per second for 5000 frames per movie. Raw data in the CFP and YFP channels was acquired by two GaASP photomultipliers (Thorlabs) separated by a dichroic filter (refl. band = 470–490 nm, trans. band = 508–675 nm). For optogenetic stimulation, a fiber optic light guide (Prizmatix) was positioned next to the objective and directed into the window to shine 590 nm light, produced by an LED driver (Doric Lenses), onto the cortex. LED power delivered using a 200 µm optical fiber was measured to be 50 µW at the cortical surface, corresponding to a power of 1.6 mW/mm^2^, which is sufficient to activate halorhodopsin^44^. Recording was carried out by an experimenter who was not blind to treatment condition.

### Novel object recognition test

Prior to behavioral testing, rats were handled and allowed 20 min of group habituation to the testing enclosure (40 cm × 40 cm) daily for 3 days. Testing was done in a well-insulated room with dimmed lights. On the day of testing, rats were allowed 7 min individual habituation, followed by 5 min exposure to familiar/familiar objects, 3 min removal to home cage, and 5 min exposure to novel/duplicate familiar objects. For rats tested a second time after D-serine treatment, a different set of objects was used. Tests were recorded using an overhead mounted digital camera (Thorlabs) and scored manually by an observer blind to treatment condition.

### Immunohistochemistry

PV-cre rats injected with AAV were transcardially perfused with ice-cold phosphate buffered saline (PBS) followed by paraformaldehyde (4% in PBS). Brains were extracted and cryoprotected in 20% then 30% sucrose before embedding in Tissue-Tek (Sakura) and sectioning with a cryostat (Leica) to obtain 30 µm thick, slide-mounted coronal sections. Sections were blocked and permeabilized for 1 h in blocking buffer (PBS with 2% normal donkey serum, 0.1% Triton X-100, and 0.01% sodium azide). Antibodies were diluted in blocking buffer and applied as follows: primary, overnight at 4°C, secondary, 2 h at room temperature. Stained slides were mounted with ProLong Antifade reagent (Invitrogen). The following antibodies and dilutions were used: rabbit anti-GFP (Millipore AB3080, 1:1000), mouse anti-Parvalbumin, (Millipore MAB1572, 1:1000), Alexa Fluor 488 goat anti-rabbit (Invitrogen, 1:400), Alexa Fluor 568 goat anti-mouse (Invitrogen, 1:400). DAPI (Roche Applied Sciences) stain was applied during post-secondary antibody wash steps following the manufacturer’s instructions. Sections were imaged using a confocal microscope (Zeiss) with identical acquisition parameters. Fluorescent intensity in unadjusted individual channels was quantified using Photoshop (Adobe).

### Data analysis

Ring-shaped ROIs were identified as previously described^24,45^. Deconvolution of ΔF/F traces obtained from these ROIs was performed using an established algorithm^31,46^ to give a spike rate estimate ʎ for each frame. Pairwise cross-correlation was computed using zero-lag correlation, and event synchronization was calculated as described previously^47^. Both were shuffle-corrected^48^ using temporally shuffled data with rate and inter-spike interval preserved^31^. Cluster, i.e. neuronal avalanche, analysis was performed as previously described^23,31^. In short, for each frame, ROIs with activity above a minimal spike rate ʎ were identified. Then ROIs in successive frames with at least one ROI active were combined into a spatiotemporal cluster to quantify cluster sizes and durations (cf. Fig. 2a) and cluster size distribution. To quantify the temporal expansion or contraction of activity over time within a cluster, we used the so-called branching parameter (ref. 25), which was calculated by dividing the total ʎ or number of active ROIs following the first frame in a cluster by the total ʎ or number of ROIs in that first frame. For behavior, object preference ratios were calculated by dividing the time spent exploring the object by the total exploration time.

### Code availability

All data analysis was carried out using custom code in Matlab (Mathworks). Matlab code is available upon request by contacting saurav.seshadri@gmail.com.

### Statistics

Individual group data was tested for normality using the Shapiro–Wilk test. Pairwise comparisons were then conducted between relevant groups using Student’s two-tailed *t*-test with Welch’s correction, unless the above test was significant, in which case the non-parametric Wilcoxon’s rank-sum test was used. Paired tests (paired *t*-test or Wilcoxon’s sign-rank test) were used when appropriate, i.e. when comparing pre- and post-drug treatment samples from the same rat, or Cont. and LED conditions for the same imaging field. Comparison of more than two independent groups was carried out using one-way ANOVA followed by Tukey’s test, unless at least one group was not normally distributed, in which case Kruskal-Wallis test was used. All statistical tests were done in Matlab (Mathworks).

## Results

### Cognitive impairment and first-order dynamics in the neonatal PCP model

The neonatal phencyclidine (PCP, an NMDAR antagonist) model is based on the NMDAR hypofunction theory of SZ^3^, and reproduces several phenotypes observed in SZ patients, ranging from neuroanatomical to behavioral^7,10^. Importantly, key neuroanatomical deficits are seen in the medial prefrontal cortex as well as M1^10^, suggesting that M1, which is accessible by minimally invasive 2PI, may show similar dynamic deficits to areas subserving working memory^49^. The main advantage of this model over acute or subchronic PCP models is its resemblance to the human disease course of SZ, in which insults during neurodevelopment produce disease onset at adolescence^20^. As a rescue paradigm, we used D-serine, an NMDAR co-agonist that has been shown to improve cognitive symptoms in SZ patients^50^ and model animals^7^.

**Fig. 1:**
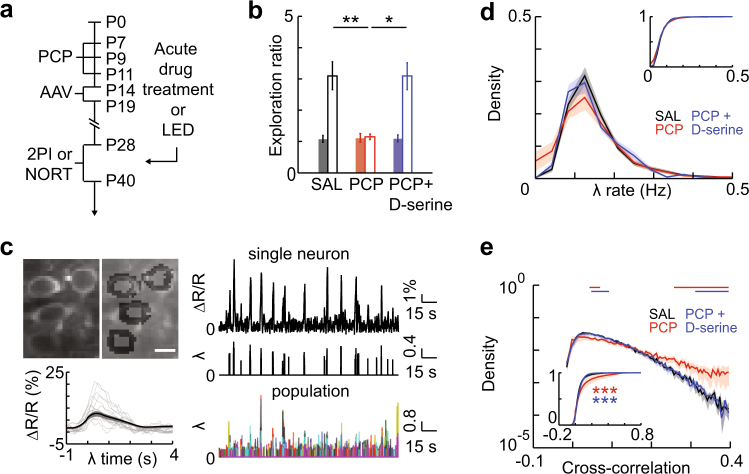
Neonatal PCP-treated rats show working memory deficits and increased pairwise cross-correlation, both rescued by D-serine. **a** Timeline of experimental procedures. **b** PCP treatment impairs novelty preference in the NORT, which is rescued by D-serine. Closed bars, familiar object exploration ratio during habituation, open bars, novel object exploration ratio. **c** Top left, representative neuronal image and segmentation, scale bar = 10 µm. Bottom left, event-centered normalized fluorescence profile; black line, average for all events in a representative movie, grey lines, example traces. Right, representative fluorescent intensity time series and thresholded spike rate estimates (λ) for a single neuron and for all neurons in a local population. **d** Firing rates are not altered by PCP treatment. Distributions of λ rates for each group. Distributions were plotted for each movie per group then averaged. Inset, mean CDFs. **e** PCP treatment shifts pairwise cross-correlation to higher values; D-serine rescues this effect. Distributions of pairwise cross-correlations for each group. Distributions were plotted for each movie per group then averaged. Bars show bins with significant group differences (Wilcoxon’s rank sum test, *P* < 0.05; red, SAL vs. PCP, blue, PCP vs. PCP + D-serine). Inset, mean CDFs. Error bars and shaded regions indicate s.e.m; *, *P* < 0.05, **, *P* < 0.01

Rats were administered PCP during early neurodevelopment, and underwent 2PI or behavioral testing at adolescence (Fig. 1a). We confirmed cognitive impairment in these rats using the novel object recognition test (NORT) of visual working memory. PCP-treated animals showed reduced preference for exploration of a novel object, compared to saline (SAL)-treated littermates (Fig. 1b, SAL, 3.10 ± 0.50, PCP, 1.15 ± 0.15, *P* = 0.002). This deficit was rescued by acute D-serine treatment (Fig. 1b, PCP + D-serine, 3.09 ± 0.68, PCP + D-serine vs. SAL, *P* = 0.701, PCP + D-serine vs. PCP, *P* = 0.031). Exploration of identical objects during the familiarization phase, as well as total exploration time, were not significantly different between groups (Fig. 1b, ratio, SAL vs. PCP, *P* = 0.971, PCP vs. PCP + D-serine, *P* = 0.742; time, Supplementary Fig. 1a). These results support the validity of this model for schizophrenia-associated cognitive dysfunction.

To study activity dynamics in local populations of pyramidal neurons, we performed in vivo 2PI of the genetically encoded calcium indicator (GECI) YC2.60 (see ref. 42). Injection of AAV expressing YC2.60 under the control of the pyramidal neuron-specific CaMKII promoter in juvenile rats (Fig. 1a) resulted in bright expression in layer 2/3 dorsal frontal and motor cortex at adolescence (Supplementary Fig. 2a,b). Imaging was conducted under light isoflurane anesthesia (1%). Imaging fields contained 80 ± 16 neurons (Supplementary Fig. 2c,d), whose pixels were grouped based on observed neuronal morphology (Fig. 1c, top left). Raw fluorescence time series extracted from identified neurons were comparable between treatment groups (Supplementary Fig. 3). For each neuron and each frame, an instantaneous firing rate estimate *λ* was obtained by standard deconvolution and thresholding^46,51^. These *λ* time series were combined to reconstruct the local network activity for the duration of the recording, which was used for further analysis (Fig. 1c).

Observed *λ* rates were within previously reported ranges^52^, and we found no change in the mean *λ* rates or distributions of rates between groups [Fig. 1d, plot shows average of within-movie distributions, *inset*, SAL vs. PCP, *P* = 0.877, SAL vs. PCP + D-serine, *P* = 0.237, PCP vs. PCP + D-serine, *P* = 0.237 (Kolmogorov-Smirnov test), Supplementary Figs. 4a-b]. Firing was uniformly irregular across groups, with a coefficient of variation in the inter-spike interval greater than one (Supplementary Fig. 4c). Thus, neonatal PCP treatment did not appear to induce any changes in first-order, cell-autonomous cortical dynamics.

### Increased pairwise cross-correlations in the neonatal PCP model

Next we studied interdependencies between neurons and asked whether neuronal pairwise cross-correlation was altered between our treatment groups. The observed values for pairwise cross-correlation were within previously reported ranges^31,52^ (Supplementary Fig. 5a). We found a significant positive shift in cross-correlations for PCP-treated rats [Fig. 1e, plot shows average of within-movie distributions, bars are bins with *P* < 0.05 (rank sum test) for SAL vs. PCP (red) or PCP vs. PCP + D-serine (blue); *inset*, SAL vs. PCP, *P* = 6.36 × 10^−8^ (Kolmogorov-Smirnov test)]. This phenotype was rescued by acute treatment with D-serine [Fig. 1e, vs. SAL, *P* = 0.08, vs. PCP, *P* = 5.59 × 10^−12^ (Kolmogorov-Smirnov test)]. The increase in cross-correlation was independent of the physical distance between neurons (Supplementary Fig. 5b). This shift in pairwise correlations towards higher values, and rescue by D-serine treatment, was also visible in the non-deconvolved fluorescence data (Supplementary Fig. 5c), and we observed an identical effect in event synchronization, an alternative measure of pairwise correlation that is independent of firing rate^47^ (Supplementary Fig. 5d). Thus, neonatal PCP treatment caused increased synchrony in ongoing cortical activity that was rescued by D-serine treatment.

### Altered avalanche dynamics in the neonatal PCP model

**Fig. 2:**
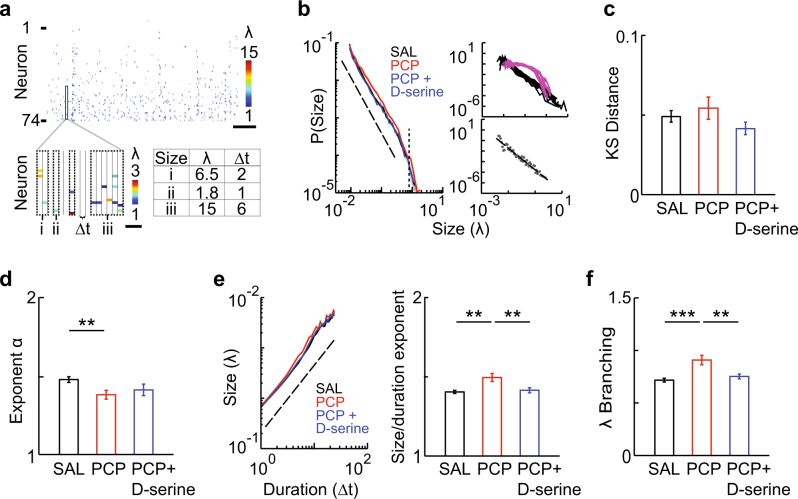
Neonatal PCP-treated rats exhibit a unique set of alterations in neuronal avalanche dynamics. **a** Spatiotemporal organization of ongoing activity. Top, raster plot of activity in a representative recording. Bottom left, expansion of boxed area showing clustering of activity (dotted lines). Bottom right, sizes and durations of example clusters. **b** Left, probability distributions of cluster sizes for each group. Distributions were plotted for each movie per group then averaged. Dotted line, power law with exponent = −1.5, dashed line, expected cutoff in size. Top right, distributions for original (black lines) and shuffled (pink lines) SAL activity rasters. Bottom right, representative binning and power law fitting. **c** PCP treatment does not produce a deviation from a power law distribution. KS distance, a statistical measure for deviation from power law distribution, for different treatment groups. **d** PCP-treated rats have shallower distributions of avalanche sizes. Power law exponents for different treatment groups. **e** PCP treatment results in faster avalanche expansion. Left, scaling of cluster size and duration for each group. Dotted line, power law with exponent = 2 as predicted from critical theory. Right, scaling exponents for different treatment groups. **f** Branching parameter for different treatment groups. Error bars and shaded regions (not always visible) indicate s.e.m; *, *P* < 0.05, **, *P* < 0.01, ***, *P* < 0.005

Cortical dynamics are known to exhibit higher-order interactions, beyond second-order correlations^53^, in the form of neuronal avalanches. Neuronal avalanches are an emergent form of network activity consisting of activity clusters whose probability distribution in sizes obeys a power law with an exponent of approximately -1.5 (see ref. 23). Clustering of ongoing activity in order to identify avalanches was carried out as previously described^31^ (Fig. 2a). As expected, cluster sizes appeared to follow power law probability distributions, which were destroyed by temporal shuffling (Fig. 2b, top right, Supplementary Fig. 6a,b). The Kolmogorov–Smirnov (KS) distance between the observed distributions and a power law was in line with previously reported values^31^, and was unchanged between groups, initially suggesting that neuronal avalanche dynamics were maintained in PCP treated rats (Fig. 2c, *P* = 0.217; Supplementary Fig. 6c). However, when the power law exponent was estimated by fitting a power law up to the predicted cutoff in size^31,54^ (Fig. 2b, bottom right), this exponent showed a significant decrease in PCP-treated rats (Fig. 2b, left, **2d**, F[3,55] = 5.04, *P* = 0.004; SAL, 1.48 ± 0.02, PCP, 1.38 ± 0.03, *P* = 0.008), indicating a shift towards larger cluster sizes over all scales. This effect was rescued by D-serine treatment (Fig. 2d, PCP + D-serine, 1.41 ± 0.04, vs. SAL, *P* = 0.428, vs. PCP, *P* = 0.894). Such a decrease in the exponent suggests an increase in synchrony, in line with the observed increase in pair-wise correlation. However, increased synchrony typically predicts a shift towards a bimodal size distribution^23^, which was not observed.

We therefore used two additional measures that specifically address temporal aspects of neuronal avalanches. Previous results and theory predict that avalanche duration and size scale with a characteristic exponent^55^. We found that this scaling exponent was significantly increased in PCP- compared to SAL-treated rats (Fig. 2e, F[3,55] = 11.48, *P* = 5.98 × 10^−6^; SAL, 1.40 ± 0.01, PCP, 1.50 ± 0.03, *P* = 0.002; Supplementary Fig. 7), but was fully rescued by acute D-serine treatment (Fig. 2e, PCP + D-serine vs. SAL, *P* = 0.969, PCP + D-serine vs. PCP, *P* = 0.006). This indicates that clusters of all durations tend to be larger in PCP-treated rats.

To identify the changes in propagation underlying such a general increase in size, we used a quantitative branching process approach^23^. Specifically, we calculated the branching parameter, a sensitive measure for the temporal evolution of a cluster (see Online Methods). We found a highly significant - more than 25% - increase in this measure for PCP-treated rats compared to SAL-treated controls (Fig. 2f, F[3,55] = 9.59, *P* = 3.44 × 10^−5^; SAL, 0.72 ± 0.02, PCP, 0.91 ± 0.04, *P* = 1.0 × 10^−4^), which was fully rescued by acute D-serine treatment (Fig. 2f, PCP + D-serine, 0.75 ± 0.02, vs. SAL, *P* = 0.826, vs. PCP, *P* = 0.002). The branching parameter in SAL-treated rats was in line with previously reported values for neuronal avalanches in vivo^31^, suggesting that this finding represents a profound alteration of avalanche dynamics in neonatal PCP-treated rats.

### Persistent bursting underlies altered avalanche dynamics in the neonatal PCP model

**Fig. 3:**
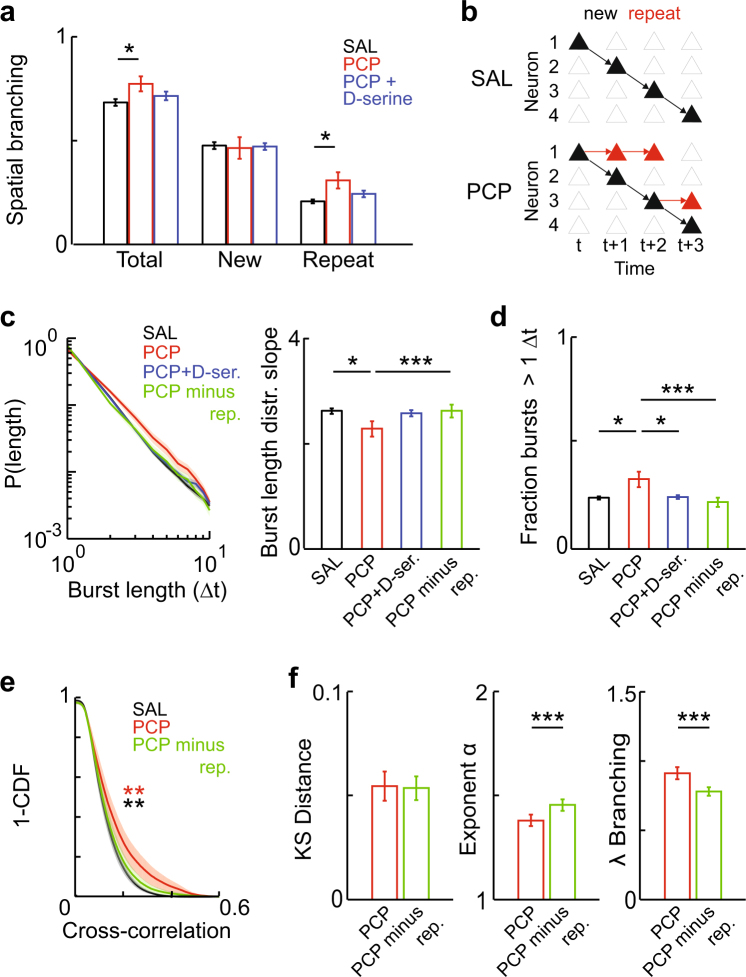
Persistent bursting in neonatal PCP-treated rats underlies abnormal spatiotemporal activity propagation. **a** PCP treatment increases spatial activity propagation specifically by increasing repeat (rather than new) neuronal activations. Branching parameter measured in number of neurons (total, new, and repeat) for different treatment groups. **b** Schematic representation of spatiotemporal activity propagation in SAL vs. PCP treated rats. Empty triangles represent quiescent neurons; filled triangles, active neurons; arrows, activity propagation. The black cascade shows a representative pattern of feedforward activity; in PCP treated rats, aberrant repeat activity (red triangles) caused by persistent bursting is superimposed on this cascade. **c** PCP-treated rats show shallower distributions in burst lengths. Left, distributions of burst lengths for different treatment groups. Distributions were plotted after pooling all bursts per movie, then averaged. Right, slope exponents for burst length distributions for different treatment groups. **d** PCP treatment leads to more persistent bursts of activity. Fraction of bursts with length greater than one frame for different treatment groups. **e** Removing persistent bursts rescues the PCP-induced increase in pairwise cross-correlation. Complementary cumulative distributions of pairwise cross-correlations for SAL, PCP, and PCP with repeat activations removed. Distributions were plotted for each movie per group then averaged. **f** KS distance, power law exponent, and branching parameter for PCP, and PCP with repeat activations removed. Error bars indicate s.e.m; *, *P* < 0.05, **, *P* < 0.01, ***, *P* < 0.001

The above results indicate that the temporal structure of avalanches is altered by PCP treatment. However, in addition to timing and amplitude of activation, the identity of active neurons is important in the representation and encoding of information by neuronal populations^56^. We therefore hypothesized that spatial activity dynamics may also be disrupted in PCP-treated rats. To address this hypothesis, we calculated the ‘spatial’ branching parameter, using the number of neurons activated during cluster propagation rather than total *λ*. We found a significant increase in spatial branching in PCP-treated rats, suggesting a wider spread of activity in the network (Fig. 3a, F[3,55] = 2.86, *P* = 0.045; SAL, 0.68 ± 0.02, PCP, 0.77 ± 0.04, *P* = 0.035). This effect was rescued by D-serine treatment (Fig. 3a, PCP + D-serine, 0.72 ± 0.02, vs. SAL, *P* = 0.705, vs. PCP, *P* = 0.222). However, further decomposition of downstream neurons into ‘new’ or ‘repeat’ activators (i.e., neurons previously active in the cluster) revealed that this effect was entirely driven by ‘repeat’ activation (Fig. 3a, new, F[3,55] = 0.23, *P* = 0.873; repeat, SAL vs. PCP, F[3,55] = 3.20, *P* = 0.030; SAL, 0.21 ± 0.01, PCP, 0.31 ± 0.04, *P* = 0.020). In other words, total spatial branching was increased not by recruitment of new neurons, but due to persistent bursting by upstream neurons (Fig. 3b). Rescue by D-serine also appeared to target repeat activation (Fig. 3a, PCP + D-serine, 0.24 ± 0.02, vs. SAL, *P* = 0.809, vs. PCP, *P* = 0.360).

Since repeat activation is comprised of bursts of activity across consecutive frames, an implication of the above finding is that burst durations of individual neurons should be increased in PCP-treated rats. We found that this was indeed the case: neuronal bursts tended to persist for longer durations in PCP-treated rats, indicated by a shallower slope of the distribution of burst lengths (Fig. 3c, F[3,55] = 2.93, *P* = 0.042; SAL, 2.62 ± 0.05, PCP, 2.29 ± 0.15, *P* = 0.04). This effect was rescued by D-serine treatment (Fig. 3c, PCP + D-serine, 2.58 ± 0.06, vs. SAL, *P* = 0.941, vs. PCP, *P* = 0.084). The overall likelihood that a burst would persist for longer than one imaging frame was significantly increased in PCP-treated rats, and fully rescued by D-serine (Fig. 3d, SAL vs. PCP, F[3,55] = 4.40, *P* = 0.008; SAL, 0.24 ± 0.01, PCP, 0.33 ± 0.04, *P* = 0.016; PCP + D-serine, 0.24 ± 0.01, vs. SAL, *P* = 0.999, vs. PCP, *P* = 0.024). These results suggest that such persistent bursting may be a key phenotype in this model.

We next examined whether this persistent bursting could be responsible for the dynamic phenotypes described above. We removed persistent bursts in movies from PCP-treated rats by randomly selecting one third of bursts longer than one frame and deleting all activity after the first frame. This intervention restored persistent bursting to levels indistinguishable from SAL-treated controls (Figs. 3c, d, PCP minus repeat, slope, 2.62 ± 0.13, *P* = 0.989; fraction, 0.22 ± 0.02, *P* = 0.423; all *P*-values vs. SAL). We found that this intervention produced pairwise cross-correlation distributions more similar to SAL-treated rats, although not identical [Fig. 3e, vs. PCP, *P* = 0.008, vs. SAL, *P* = 0.005 (Kolmogorov-Smirnov test)]. However, it fully rescued the size distribution exponent, size/duration scaling, and branching phenotypes without affecting KS distance (Fig. 3f, Supplementary Fig. 8, KS distance, *P* = 0.934; exponent, 1.45 ± 0.03, *P* = 1.22 × 10^−4^; scaling, 1.46 ± 0.02, *P* = 6.10 × 10^−5^; branching, 0.78 ± 0.03, *P* = 6.10 × 10^−5^; spatial branching, 0.68 ± 0.04, *P* = 6.10 × 10^−5^; all *P*-values vs. PCP). These results demonstrate that persistent bursting is the underlying cause for the range of dynamic phenotypes observed in this model. Furthermore, they suggest that D-serine may rescue cognitive performance by specifically reversing this persistent bursting phenotype.

### Altered avalanche dynamics are mediated by GABAergic signaling, but are not induced by acute inhibition of PVins

**Fig. 4:**
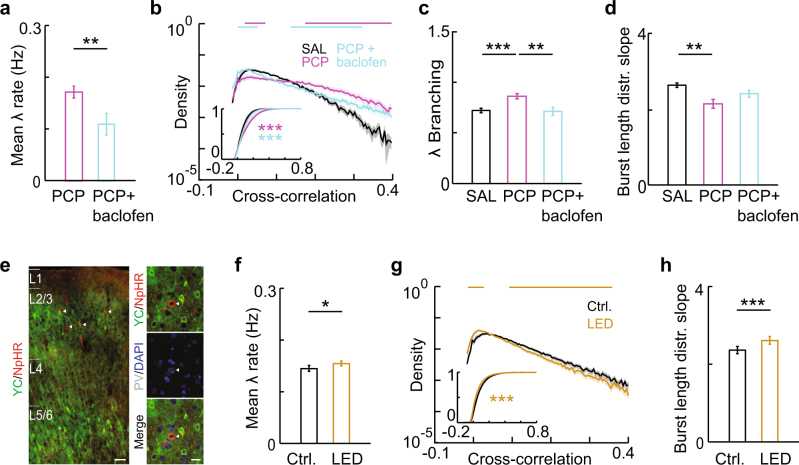
Neonatal PCP treatment-induced changes in avalanche dynamics are rescued by enhancing GABAergic signaling, but not reproduced by inhibiting PVins. **a** Baclofen inhibts network activity. Mean *λ* rates for each group. **b** Baclofen rescues the PCP-induced increase in pairwise cross-correlation. Distributions of pairwise cross-correlations for each group. Distributions were plotted for each movie per group then averaged. Bars show bins with significant group differences (Wilcoxon’s rank sum test, *P* < 0.05; magenta, SAL vs. PCP, cyan, PCP vs. PCP + baclofen). Inset, mean CDFs. **c** Branching parameter for different treatment groups. **d** Baclofen rescues PCP-induced persistent bursting. Slope exponents for burst length distributions for different treatment groups. **e** YC2.60, NpHR3.0, and PV labeling in AAV-injected PV-Cre rats. Scale bar, left, 100 µm. right, 20 µm. **f** PVin inhibition increases network activity. Mean λ rates from movies of identical regions without (Ctrl.) and with LED stimulation. **g** PVin inhibition decreases pairwise cross-correlations. Distributions of pairwise cross-correlations for both stimulation groups. Distributions were plotted for each movie per group then averaged. Bars show bins with significant group differences (Wilcoxon’s rank sum test, *P* < 0.05). **h** PVin inhibition produces steeper distributions in burst lengths. Slope exponents for burst length distributions for both stimulation groups. Error bars and shaded regions indicate s.e.m; *, *P* < 0.05

To understand the role of interneuronal dysfunction in producing these phenotypes, we attempted to rescue them by potentiating inhibitory neurotransmission. We used baclofen, a GABA_B_ receptor agonist that has previously been shown to rescue network excitatory-inhibitory balance and working memory dysfunction in a mouse developmental NMDAR hypofunction model^40^. We observed that systemic injection of baclofen reduced average firing rates (Fig. 4a, F[3,49] = 8.24, *P* = 1.53 × 10^−4^; PCP, 0.17 ± 0.01; PCP + baclofen, 0.11 ± 0.02; *P* = 0.004), and rescued the increases in pairwise cross-correlations [Fig. 4b, plot shows average of within-movie distributions, *n* = 12 per group, bars are bins with *P* < 0.05 (rank sum test) for SAL vs. PCP (magenta) or PCP vs. PCP + baclofen (cyan)], branching parameter (Fig. 4c, F[3,49] = 6.36, *P* = 9.95 × 10^−4^; PCP, 0.86 ± 0.03; PCP + baclofen, 0.71 ± 0.04; PCP vs. SAL, *P* = 0.004, PCP vs. PCP + baclofen, *P* = 0.003), and persistent bursting (indicated by a decrease in the burst length distribution slope, Fig. 4d, F[3,49] = 3.99, *P* = 0.013; PCP, 2.02 ± 0.15; PCP + baclofen, 2.47 ± 0.15; PCP vs. SAL, *P* = 0.016, PCP vs. PCP + baclofen, *P* = 0.035) that were reproducibly induced by PCP treatment in a second, independent cohort of rats. Notably, the spatial branching parameter also showed an increase that was driven by repeat activations, and was accompanied by changes in the slope exponent and size/duration scaling that matched those observed in PCP-treated rats (Supplementary Fig. 9). These results confirm that inhibitory GABAergic signaling underlies the specific dynamic phenotypes observed in neonatal PCP model animals.

We next sought to test the causal role of PVin dysfunction in the alterations of avalanche dynamics observed in PCP-treated rats. AAV with Cre-dependent expression of the inhibitory opsin NpHR3.0 (see ref. 57) was co-injected with YC2.60 in juvenile PV-Cre rats. This resulted in sparse opsin expression specifically in layer 2/3 PVins (23 ± 4% of PV-positive cells), in parallel with pyramidal neuron-specific GECI expression at adolescence (Fig. 4e). LED-driven stimulation of this opsin is expected to reduce PVin activity and elevate pyramidal neuron spiking^58,59^, functionally mimicking network disinhibition produced by reduced PVin density, which has been reported in the neonatal PCP model^10^. Consistent with this prediction, we observed increased mean firing rates during LED stimulation (Fig. 4f, Ctrl., 0.14 ± 0.006, LED, 0.15 ± 0.005, *P* = 0.011). Opsin stimulation also led to a significant decrease in pairwise cross-correlations [Fig. 4g, plot shows average of within-movie distributions, *n* = 20 per group, bars are bins with *P* < 0.05 (sign rank test) for Ctrl. vs. LED], which is in line with expectations that reducing perisomatic PVin inhibition decreases spike timing precision in pyramidal neurons, thus decreasing network synchrony^60^. This overall reduction in synchrony is contrary to the increase in synchrony observed in PCP-treated rats (Fig. 1e). PVin inhibition did not reproduce the altered spatial branching, size distribution exponent, or branching parameter phenotypes observed in PCP-treated rats (Supplementary Fig. 10a-d, spatial branching, *P* = 0.654; exponent, *P* = 0.852; branching parameter, *P* = 0.167), and in fact produced a decrease in persistent bursting (indicated by a steeper distribution of burst lengths, (Fig. 4h, Ctrl., 2.36 ± 0.08, LED, 2.61 ± 0.09, *P* = 0.002).

## Discussion

In the present study, we identify a novel dynamic phenotype at the microcircuit level in a neurodevelopmental NMDAR hypofunction model (neonatal PCP). 2PI of local groups of cortical pyramidal neurons revealed a shift towards increased pairwise cross-correlation, and significant alterations in neuronal avalanche dynamics. These alterations could largely be traced to a persistent bursting phenotype, which produced aberrant, non-feedforward spatial and temporal propagation of activity in PCP-treated rats. Importantly, these phenotypes were rescued by acute treatment with D-serine, an NMDAR co-agonist, or baclofen, a GABA_B_ receptor agonist, administered under conditions which also rescue working memory performance. This finding strongly suggests that critical dynamics facilitate cognition, and that altered avalanche dynamics are pathological state-specific markers. This in turn suggests that the phenotypes described here could be useful for diagnosis or therapeutic screening for cognitive impairment associated with NMDAR hypofunction.

Interestingly, the phenotypes we observed were not reproduced by optogenetically inhibiting PV-positive interneurons, suggesting that loss-of-function of these cells does not contribute to NMDAR hypofunction-associated network dysfunction. This lack of an effect on higher-order interactions was surprising given that PV interneuron inhibition acutely disinhibited and desynchronized the network, as previously reported^19,58,59^. Our observations of several significant changes in the opposite direction to those observed in PCP-treated rats suggest that the negative result was not due to insufficient PVin inhibition. One possible explanation is that non-fast-spiking interneurons, expressing the marker somatostatin (SOMins), mediate NMDAR hypofunction-induced excitatory/inhibitory imbalance. SOMins show stronger NMDAR expression and currents^61^, and also affect pyramidal neuron excitability^62^, as well as bursting activity in pyramidal neurons^63,64^, an effect consistent with our observation of increased burst duration in PCP-treated rats.

The propagation phenotype identified here did not include an outright deviation from criticality, representing a qualitative difference from phenotypes observed in vitro following alteration of excitatory/inhibitory balance. In those experiments, pharmacological disinhibition of the network simultaneously increased the branching parameter and shifted the power law in size distributions towards a bimodal distribution, a mark of supercritical dynamics^25–27^. In fact, the phenotype observed here was more similar to that seen following in vivo and in vitro modulation of dopaminergic signaling, in which power law distributions of avalanches are preserved, but exponents vary following an inverted-U shaped profile^27,28^. These changes may be specific to psychiatric illness, as epilepsy, another disorder of altered excitatory/inhibitory balance, shows a more straightforward supercritical phenotype^65^. Further investigation of these mechanisms, using avalanche dynamics as a quantitative readout, may therefore provide insight into the unique pathophysiology of SZ.

The precise link between altered dynamics described above and impaired cognition, such as working memory, remains to be determined. Recent modeling work has shown that reduced NMDAR signaling to interneurons can produce broadened spatial representations, resulting in impaired working memory performance^66^. The increased pairwise cross-correlation we observed in PCP-treated rats is consistent with this model of impairment. Previous work from our group suggests that feedforward propagation is characteristic of avalanche dynamics and non-feedforward propagation is rare^54^. A recent computational model proposed sequential neuronal activation as a substrate for short-term memory, which would be disrupted by non-feedforward activity^67^. Finally, excessive bursting, as we observed, could contribute to noisy sensory integration^64^, which is observed in SZ and associated with clinical measures^68^.

To our knowledge, no studies have directly measured critical dynamics in patients with SZ; however, there are several ways to integrate our current findings with existing data characterizing abnormal cortical dynamics in SZ patients using electrophysiology. Elevated cross-correlations at the neuronal level in cortical networks, such as we observed, could underlie increased resting oscillatory power^69^ and coherence^70^ seen in SZ patients by EEG recording. Our finding that these increased correlations are largely a byproduct of persistent bursting by individual neurons, rather than a true increase in connectivity, is consistent with reduced dendritic spine density^71^ and excitatory neurotransmission^72^ in SZ, and with observations that evoked gamma power is impaired in SZ patients^69^. The altered temporal propagation we describe could reflect impaired phase-locking of neuronal firing with oscillations, which is thought to facilitate information encoding by organizing the precise timing and sequential procession of neuronal activity^73^. This mechanism is likely disrupted along with cross-frequency phase coupling in SZ patients^74^. While further work combining 2PI and EEG recording is necessary, our results provide an indication of how this disruption may manifest at the neuronal level. In addition to EEG, a wealth of functional and structural neuroimaging has provided insight into network dysfunction in SZ patients. DTI analysis shows that SZ patients have elevated connectivity in frontal cortical networks^75^, and resting state fMRI shows hyperactivity and hyperconnectivity within the default mode network in SZ^76^. Similarly, NMDAR antagonist treatment produces working memory deficits partly by impairing suppression of default mode network activity, and BOLD signal modeling suggests that these effects can be attributed to reduced GABAergic inhibition^77^. Though it is difficult to draw direct comparisons between neuronal firing and BOLD fluctuations, these findings are strikingly similar to our observations of persistent bursting at the neuronal level, which was rescued by administration of the GABA receptor agonist baclofen. Finally, the impaired feedforward propagation of activity we report could contribute to altered network topology in SZ, including reduced clustering and small-worldness revealed by graph theoretical analysis of imaging data from SZ patients^78^. Moving forward, hypothesis-driven studies that combine population recording (fMRI or EEG) with modulation of neuronal activity (e.g., using optogenetics), guided by disease-associated phenotypes as reported here, could be invaluable to testing the links proposed above.

The notion that avalanche dynamics influence cognition is in line with a recent human neuroimaging study which demonstrated that fluctuations in neuronal avalanches closely track inter-individual variability in cognitive scaling laws^34^. In recent years, the framework of neuronal avalanches has been used to describe network alterations in sleep deprived^33^ and epileptic^79^ cortical networks. Overall, we believe that our results support a conceptual shift towards using high-resolution, quantitative analysis of network dynamics, particularly higher-order interactions, to gain insight into potentially disease-related pathophysiology.

## Electronic supplementary material

Supplementary Figures 1-11
