# Supplementary material for: Altered avalanche dynamics in a developmental NMDAR hypofunction model of cognitive impairment
Source: Transl Psychiatry. 2018 Jan 10;8:3. doi: 10.1038/s41398-017-0060-z (PMC5802445; doi:10.1038/s41398-017-0060-z)
Supplement: Supplementary file 1 — Supplementary Figures 1-11 [file 41398_2017_60_MOESM1_ESM.pdf]

Seshadri et. al.
Supplementary Figure 1

a

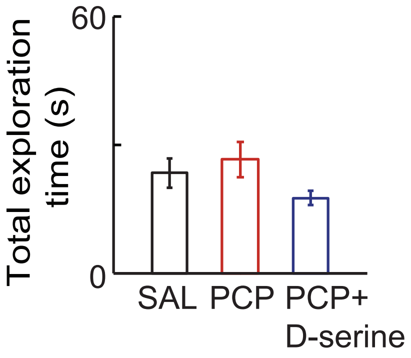

Supplementary Fig. 1: Object exploration control for NORT. a) Total exploration time during the test phase was not significantly different between treatment groups (SAL vs. PCP, p = 0.7650, PCP vs. PCP+D-serine, p = 0.219). Error bars indicate s.e.m.

Seshadri et. al.
Supplementary Figure 2

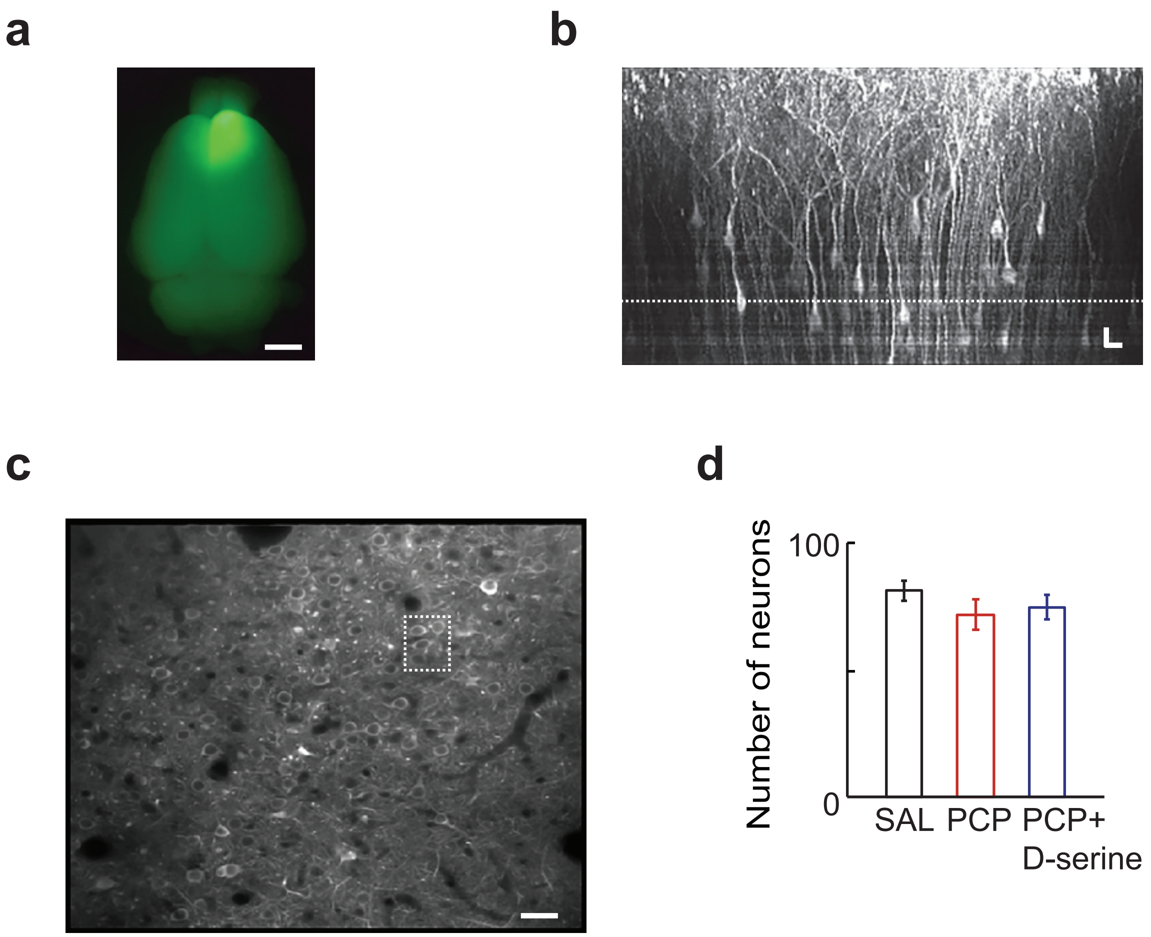

Supplementary Fig. 2: YC2.60 expression in layer 2/3 pyramidal neurons. a) Low magnification image of whole brain extracted from adolescent rat injected with AAV expressing YC2.60 in dorsal frontal cortex (green). Scale bar: 1 cm. b) Side projection of a z-stack showing YC2.60 expression in cells with characteristic pyramidal neuron morphology. Dashed line shows representative imaging plane. Scale bar: vertical, 30 μm, horizontal, 30 μm. c) Widespread expression of YC2.60 in pyramidal neurons in a representative imaging field. Boxed area is enlarged in Fig. 1c. Scale bar: 30 μm. d) Number of neurons per imaging field was not significantly different between treatment groups ($F(3,55) = 1.32$, $p = 0.278$). Error bars indicate s.e.m.

Seshadri et. al.
Supplementary Figure 3

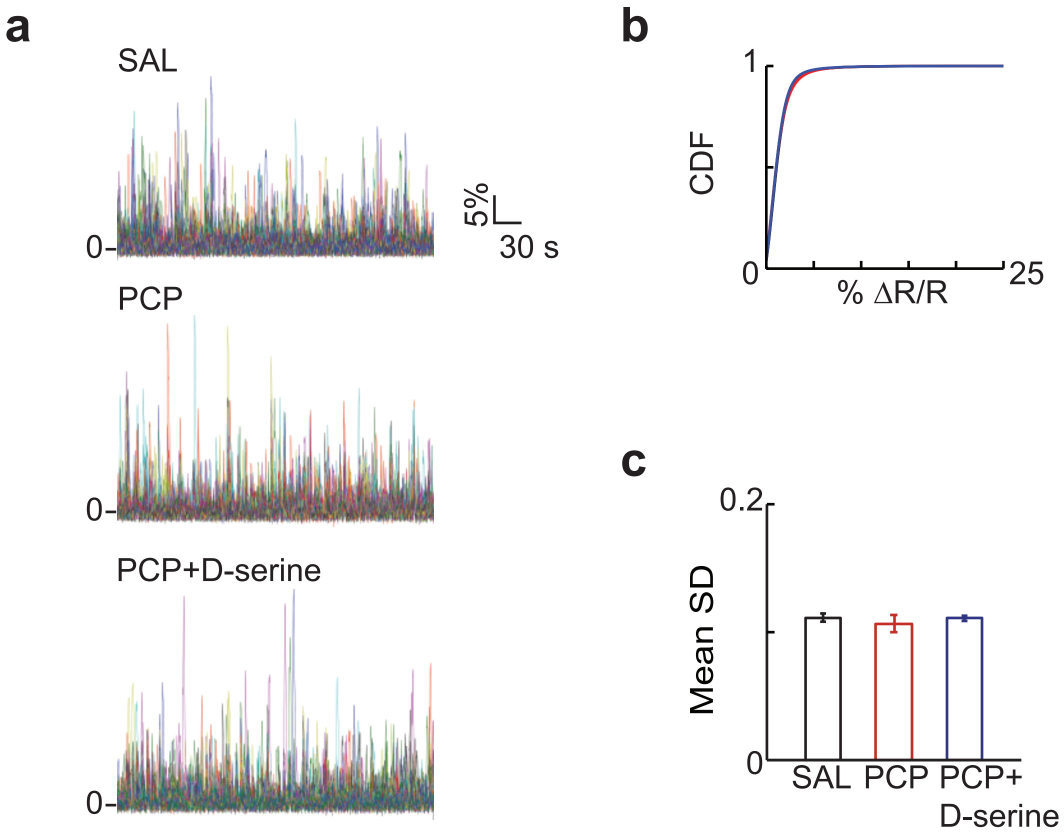

Supplementary Fig. 3: Consistency of in vivo recordings. a) Fluorescent intensity recordings for different drug treatment groups. ΔR/R traces from individual ROIs are color-coded and overlaid for representative SAL, PCP, and PCP+D-serine recordings. b) Distributions of ΔR/R values were not significantly different between groups. CDFs were plotted for each movie per group then averaged. Shaded regions (not visible) indicate s.e.m. c) Mean standard deviation in ΔR/R was not significantly different between groups ($F(3,55) = 0.53$, $p = 0.660$). Error bars indicate s.e.m.

Seshadri et. al.
Supplementary Figure 4

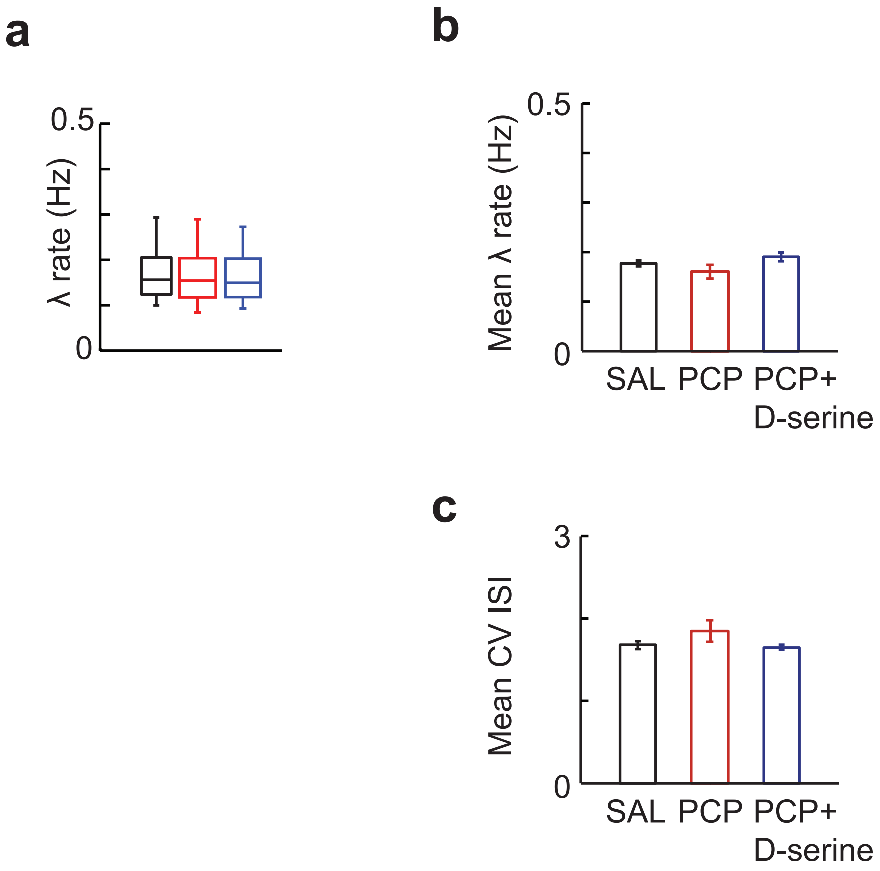

Supplementary Fig. 4: First-order dynamic phenotypes. a) Box plots of λ rates (all neurons pooled per group). No significant differences were observed between treatment groups (One-way ANOVA, rate, $F(3,4517) = 0.42$, $p = 0.736$; SAL, 0.191 ± 0.015 Hz, N = 1,296 neurons from 6 rats; PCP, 0.176 ± .004 Hz, N = 1,146 neurons from 6 rats; PCP + D-serine, 0.191 ± 0.011 Hz, N = 1,117 neurons from 4 rats) b) Average λ rate was not significantly different between groups ($H(3,55) = 3.85$, $p = 0.279$). c) Average coefficient of variation in the inter-spike interval (CV ISI) was not significantly different between groups. ($F(3,55) = 1.40$, $p = 0.253$). Error bars, s.e.m.

Seshadri et. al.
Supplementary Figure 5

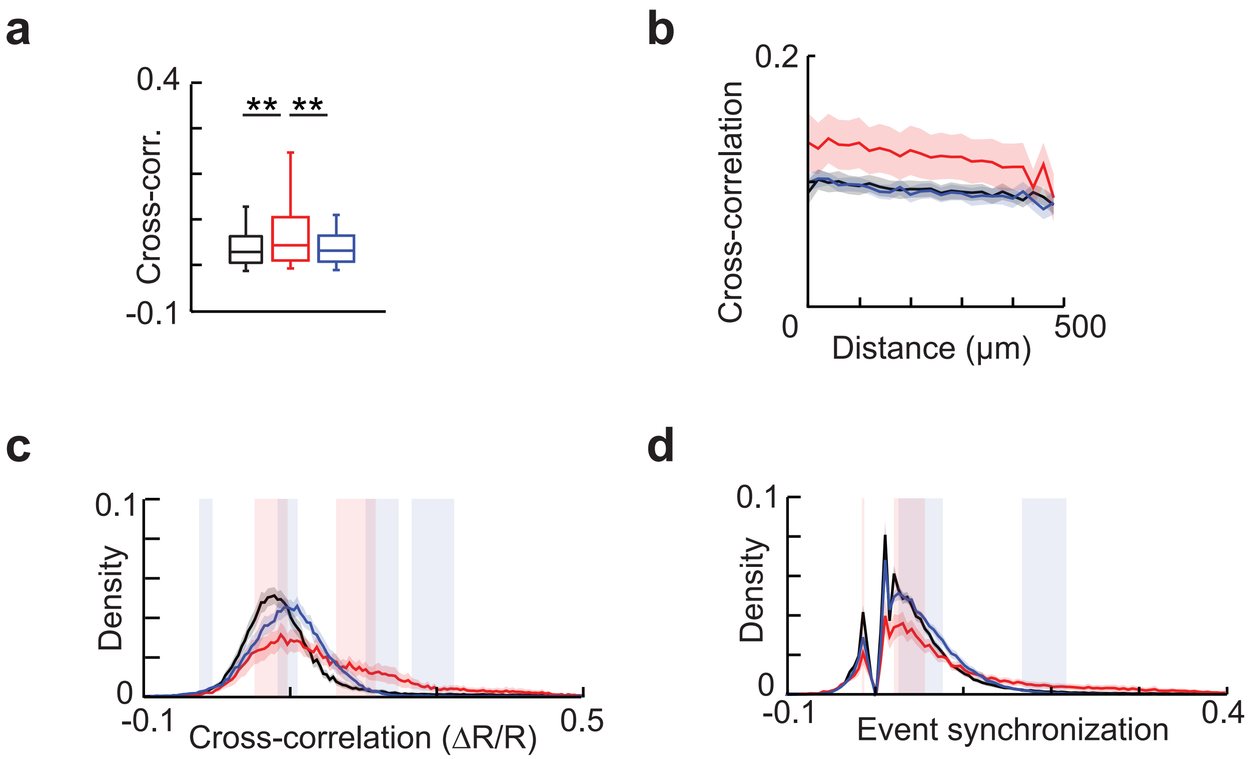

Supplementary Fig. 5: Second-order dynamic phenotypes. a) Box plots of cross-correlations (all pairs pooled per group). PCP-treated rats showed a significant increase in pairwise cross-correlations, which was rescued by D-serine treatment (F(3, 1.48x105) = 546.29, p < 1x10-10 ; SAL, 0.098 ± 3.2x10-4, N = 53,618 pairs, PCP, 0.115 ± 4.3x10-4, N = 44,702 pairs, p = 3.77x10-9, PCP+D-serine, 0.095 ± 3.2x10-4, N = 43,448 pairs, vs. SAL, p = 5.55x10-9, vs. PCP, p = 3.77x10-9) b) Dependence of pairwise cross-correlation on distance between pairs of neurons. c) Distributions of pairwise cross-correlations in raw ΔR/R for different treatment groups. Distributions were plotted for each movie per group then averaged. Vertical shaded regions show bins with significant group differences (Wilcoxon's rank sum test, p<0.05; red, SAL vs. PCP, blue, PCP vs. PCP+D-serine). d) Distributions of pairwise event synchronization for different treatment groups. Distributions were plotted for each movie per group then averaged. Vertical shaded regions show bins with significant group differences (Wilcoxon's rank sum test, p<0.05; red, SAL vs. PCP, blue, PCP vs. PCP+D-serine). Error bars and shaded regions indicate s.e.m; **, p<0.01.

Seshadri et. al.
Supplementary Figure 6

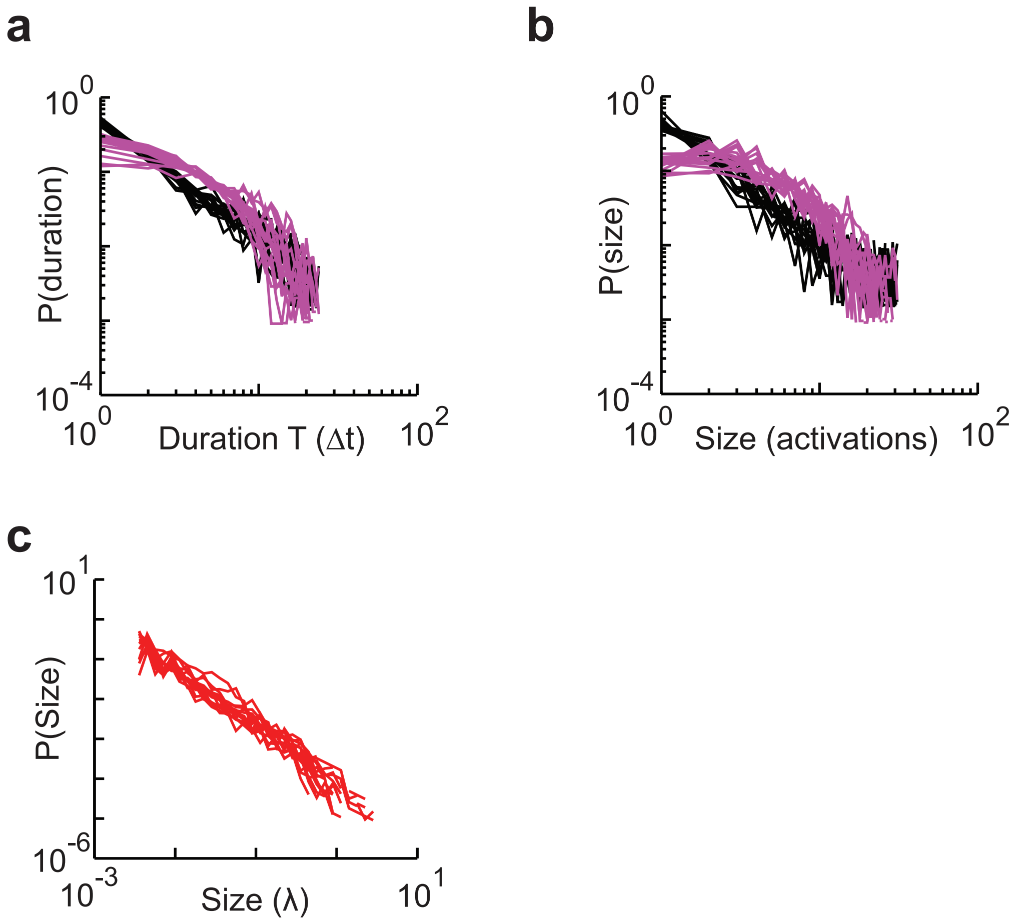

Supplementary Fig. 6: Temporal shuffling destroys power law distributions in sizes. a) Size probability distributions for original (black lines) and shuffled (pink lines) SAL activity rasters, with size measures in number of frames. b) Size probability distributions for original (black lines) and shuffled (pink lines) SAL activity rasters, with size measures in number neuronal activations. c) Individual size probability distributions for PCP-treated rats. Distributions do not exhibit obvious deviations from a power law, such as bimodality.

Seshadri et. al.
Supplementary Figure 7

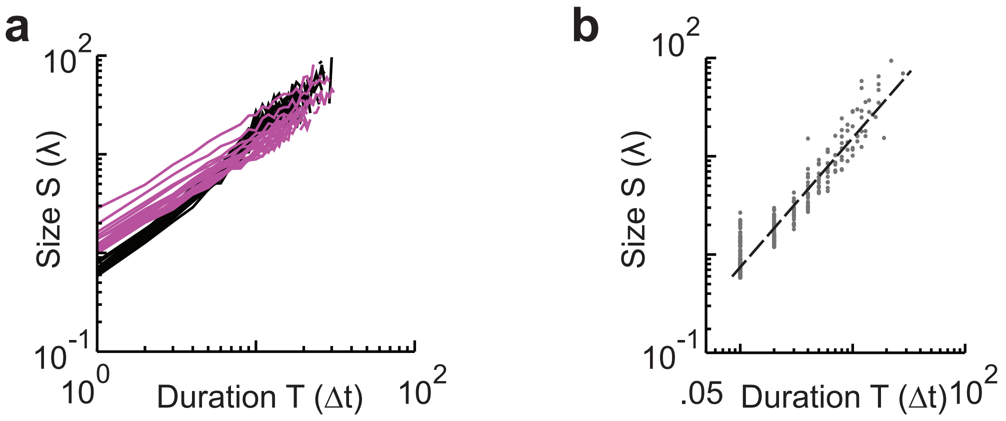

Supplementary Fig. 7: Temporal shuffling changes scaling of size and duration. a) Scaling of size and duration for original (black lines) and shuffled (pink lines) SAL activity rasters. b) Example of power law fitting to scaling distribution for quantification of slope exponent.

Seshadri et. al.
Supplementary Figure 8

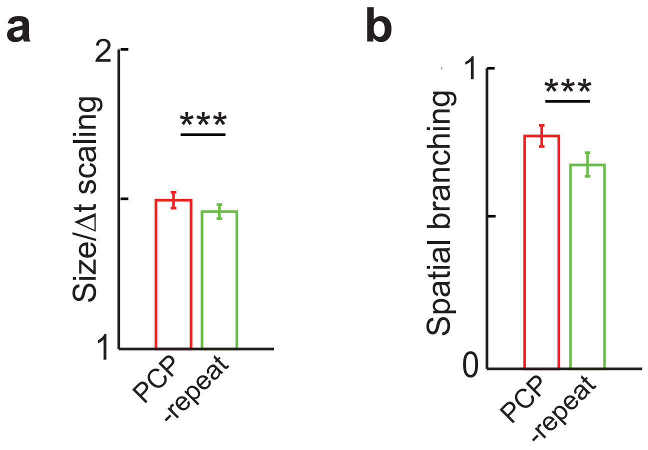

Supplementary Fig. 8: Removing persistent bursting rescues neuronal avalanche phenotypes. a) Size/lifetime scaling exponent for PCP and PCP with repeat activations removed. b) Spatial branching parameter for PCP and PCP with repeat activations removed.

Seshadri et. al.
Supplementary Figure 9

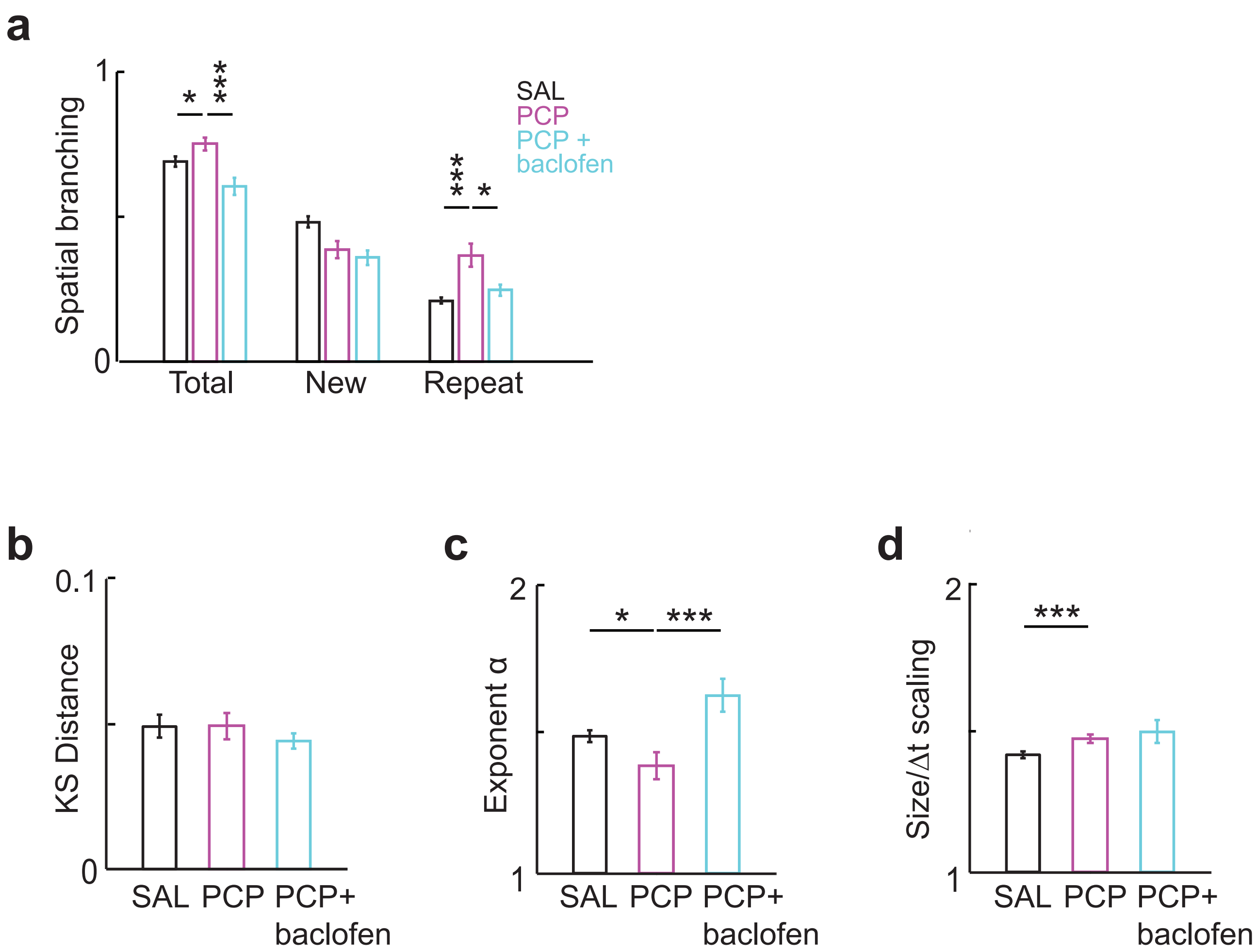

Supplementary Fig. 9: Baclofen treatment rescued PCP-induced avalanche phenotypes. a) Branching parameter measured in number of neurons (total, new, and repeat) for different treatment groups. b) KS distance for different treatment groups. c) Power law exponents for cluster size probability distributions for different treatment groups. d) Branching parameter for different treatment groups. Error bars indicate s.e.m; *, p< 0.05, ***, p<0.005.

Seshadri et. al.
Supplementary Figure 10

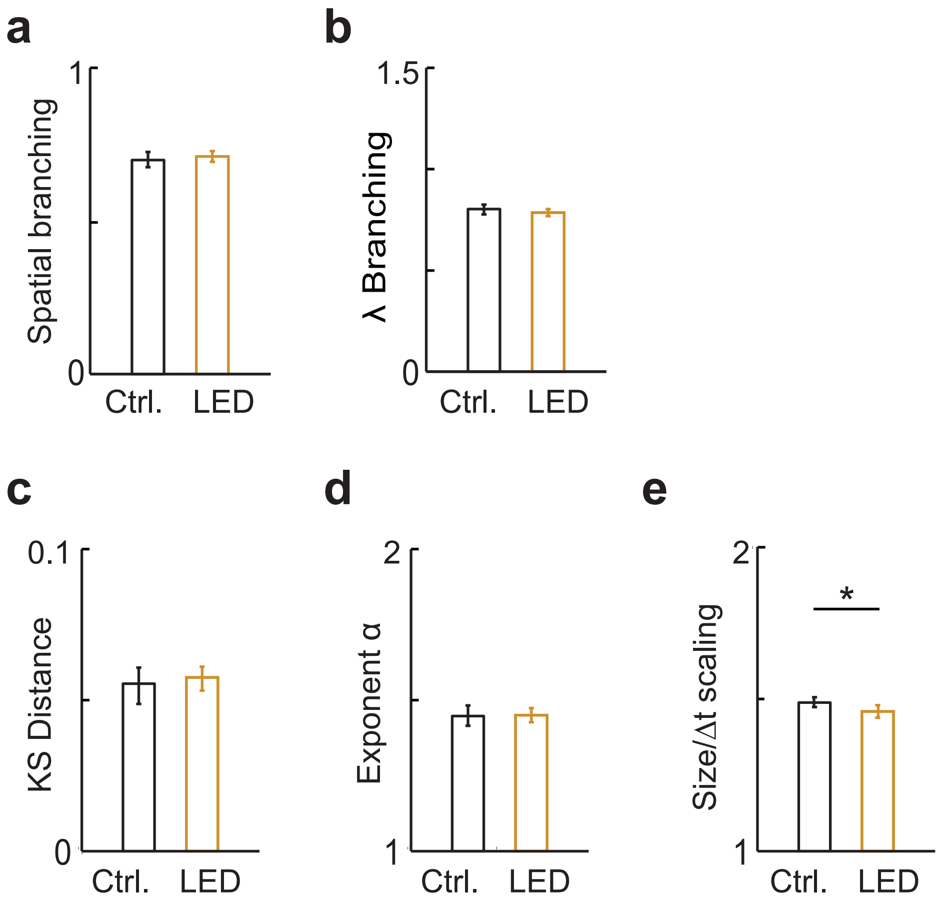

Supplementary Fig. 10: FSIN inhibition did not replicate PCP-induced avalanche phenotypes. a) Spatial branching parameter for both stimulation groups. b) Branching parameter for both stimulation groups. c) KS distance for both stimulation groups. d) Power law exponents for cluster size probability distributions for both stimulation groups. e) Size and duration scaling exponents for both stimulation groups (Cont., 1.49 ± 0.02, LED, 1.46 ± 0.02, $p = 0.048$). Error bars indicate s.e.m; *, $p < 0.05$, **, $p < 0.01$.

Seshadri et. al.
Supplementary Figure 11

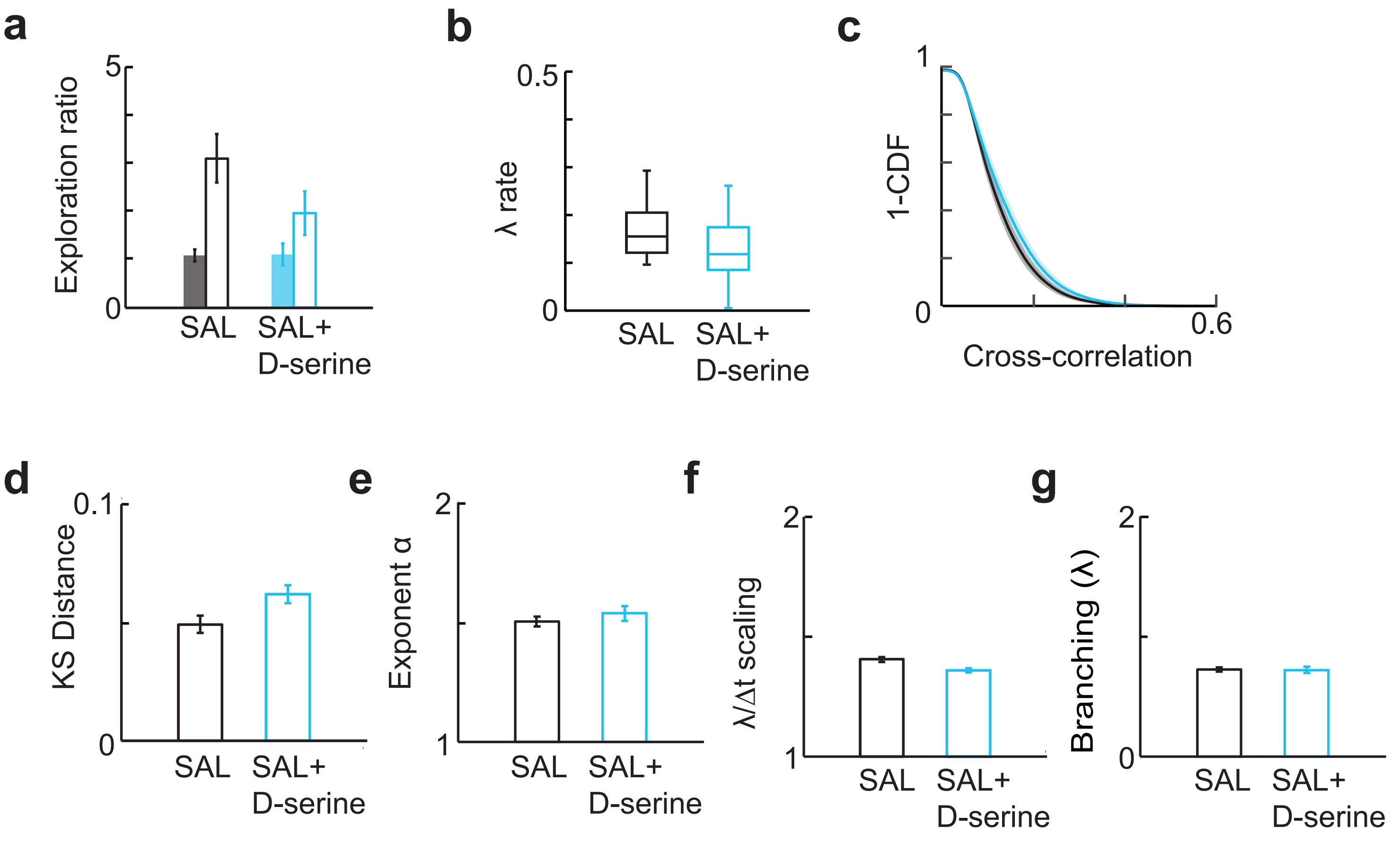

Supplementary Fig. 11: Key results for SAL+D-serine treatment group. No significant differences were observed between SAL and SAL+D-serine treatment groups in several key parameters, for which PCP and PCP+D-serine showed clear differences. a) NORT performance. b) λ rate. c) Pairwise cross-correlation. d) KS distance. E) Power law exponent. f) Scaling of size and duration. g) Branching parameter.
